# Low-flow time and outcomes in hypothermic cardiac arrest patients treated with extracorporeal cardiopulmonary resuscitation: a secondary analysis of a multi-center retrospective cohort study

**DOI:** 10.1186/s40560-024-00735-1

**Published:** 2024-06-11

**Authors:** Kosuke Shoji, Hiroyuki Ohbe, Tasuku Matsuyama, Akihiko Inoue, Toru Hifumi, Tetsuya Sakamoto, Yasuhiro Kuroda, Shigeki Kushimoto, the SAVE-J II study group, Hirotaka Sawano, Yuko Egawa, Shunichi Kato, Kazuhiro Sugiyama, Naofumi Bunya, Takehiko Kasai, Shinichi Ijuin, Shinichi Nakayama, Jun Kanda, Seiya Kanou, Toru Takiguchi, Shoji Yokobori, Hiroaki Takada, Kazushige Inoue, Ichiro Takeuchi, Hiroshi Honzawa, Makoto Kobayashi, Tomohiro Hamagami, Wataru Takayama, Yasuhiro Otomo, Kunihiko Maekawa, Takafumi Shimizu, Satoshi Nara, Michitaka Nasu, Kuniko Takahashi, Yoshihiro Hagiwara, Reo Fukuda, Takayuki Ogura, Shinichiro Shiraishi, Ryosuke Zushi, Norio Otani, Migaku Kikuchi, Kazuhiro Watanabe, Takuo Nakagami, Tomohisa Shoko, Nobuya Kitamura, Takayuki Otani, Yoshinori Matsuoka, Masaaki Sakuraya, Hideki Arimoto, Koichiro Homma, Hiromichi Naito, Shunichiro Nakao, Tomoya Okazaki, Yoshio Tahara, Hiroshi Okamoto, Jun Kunikata, Hideto Yokoi

**Affiliations:** 1 Division of Emergency and Critical Care Medicine, Tohoku University Graduate School of Medicine, 1-1, Seiryo-Machi, Aoba-ku, Sendai, Miyagi 9808574 Japan; 2 Department of Emergency Medicine, Japanese Red Cross Ishinomaki Hospital, Ishinomaki, Japan; 3 Department of Emergency Medicine, Kyoto Prefectural University of Medicine, Kyoto, Japan; 4 Department of Emergency and Critical Care Medicine, Hyogo Emergency Medical Center, Kobe, Japan; 5 Department of Emergency and Critical Care Medicine, St. Luke’s International Hospital, Tokyo, Japan; 6 Department of Emergency Medicine, Teikyo University School of Medicine, Tokyo, Japan; 7 Department of Emergency Medicine, Kagawa University School of Medicine, Miki, Kagawa Japan

**Keywords:** Accidental hypothermia, Extracorporeal cardiopulmonary resuscitation, Low-flow time, Out-of-hospital cardiac arrest

## Abstract

**Background:**

In out-of-hospital cardiac arrest (OHCA) patients with extracorporeal cardiopulmonary resuscitation (ECPR), the association between low-flow time and outcomes in accidental hypothermia (AH) patients compared to those of patients without AH has not been fully investigated.

**Methods:**

This was a secondary analysis of the retrospective multicenter registry in Japan. We enrolled patients aged ≥ 18 years who had been admitted to the emergency department for OHCA and had undergone ECPR between January, 2013 and December, 2018. AH was defined as an arrival body temperature below 32 °C. The primary outcome was survival to discharge. Cubic spline analyses were performed to assess the non-linear associations between low-flow time and outcomes stratified by the presence of AH. We also analyzed the interaction between low-flow time and the presence of AH.

**Results:**

Of 1252 eligible patients, 105 (8.4%) and 1147 (91.6%) were in the AH and non-AH groups, respectively. Median low-flow time was 60 (47–79) min in the AH group and 51 (42–62) min in the non-AH group. The survival discharge rates in the AH and non-AH groups were 44.8% and 25.4%, respectively. The cubic spline analyses showed that survival discharge rate remained constant regardless of low-flow time in the AH group. Conversely, a decreasing trend was identified in the survival discharge rate with longer low-flow time in the non-AH group. The interaction analysis revealed a significant interaction between low-flow time and AH in survival discharge rate (*p* for interaction = 0.048).

**Conclusions:**

OHCA patients with arrival body temperature < 32 °C who had received ECPR had relatively good survival outcomes regardless of low-flow time, in contrast to those of patients without AH.

**Supplementary Information:**

The online version contains supplementary material available at 10.1186/s40560-024-00735-1.

## Background

Accidental hypothermia (AH) is an involuntary drop in core body temperature to < 35 °C. At a core body temperature below 32 °C, there is a risk of cardiac arrest, and the risk becomes significantly elevated below 28 °C [1]. Previous nationwide and international studies of AH have reported mortality rates of 12.0–26.9% [2–4]. The incidence and mortality rates of AH are higher in geriatric patients, and the number of AH patients is predicted to rise in coming decades [5]. Therefore, AH treatment is a matter of medicine and public health.

Extracorporeal cardiopulmonary resuscitation (ECPR), which applies extracorporeal membrane oxygenation (ECMO) to patients with cardiac arrest refractory to conventional cardiopulmonary resuscitation (CPR), has been reported to improve outcomes for AH patients presenting with cardiac arrest or circulation instability [6–9]. Previous studies have also reported cases of prolonged cardiac arrest with hypothermia who received ECPR, in which the patients survived without neurologic impairment [10–12].

Previous studies have revealed that low-flow time, the duration between the initiation of conventional CPR and the implementation of ECPR, is associated with ECPR outcomes for cardiac arrest patients [13–17]. The guidelines from the European Resuscitation Council and the Extracorporeal Life Support Organization recommend initiating ECPR until 60 min of low-flow time [18, 19]. However, whether ECPR for cardiac arrest patients with AH results in good neurological outcomes even with prolonged low-flow time has not yet been fully investigated. Consequently, the present study aimed to assess the association between low-flow time and outcomes in AH patients and compared to those of patients without AH among a cohort of OHCA patients resuscitated using ECPR.

## Methods

### Study design and data

This study was a secondary analysis of the multicenter retrospective cohort study, the Study of Advanced life support for Ventricular fibrillation with Extracorporeal circulation in Japan II (SAVE-J II) in Japan. Thirty-six institutions in Japan participated in this registry. This multicenter study was pre-registered at the Japanese clinical trial registry (registration number: UMIN000036490) [20], and approved by the institutional review board of Kagawa University (approval number: 2018-110) and each participating institution. Owing to the retrospective study design, informed consent was not required. However, patients were given the opportunity to opt out of the study at any time by withdrawing permission to use their data. To ensure patients’ right to refuse participation, the methods of utilizing patient information were disclosed on the websites and notice boards of each participating institution.

SAVE-J II included all patients aged ≥ 18 years who were admitted to the emergency department for OHCA and underwent ECPR between January 1, 2013 and December 31, 2018. In this registry, ECPR was defined as resuscitation for cardiac arrest using veno-arterial ECMO. The exclusion criteria were patients with in-hospital cardiac arrest and refusal to participate in the study communicated by the patients themselves, family, or others. The following data were collected: patient characteristics, prehospital information, information on hospital arrival, diagnosis, interventions, mechanical support information, time course, and outcomes [21].

### Study population and data collection

We included all patients of the SAVE-J II study. The exclusion criteria of this secondary analysis were as follows: return of spontaneous circulation before ECMO pump on; transfer from another hospital; and missing data on outcomes (survival to hospital discharge and cerebral performance category [22] at hospital discharge), low-flow time, body temperature at hospital arrival, and covariates, as mentioned below.

The following patient data were used for this secondary analysis: age, sex, location of cardiac arrest (home, public space, or ambulance), witnessed cardiac arrest, bystander CPR, initial cardiac rhythm (shockable or unshockable) at the scene and at hospital arrival, body temperature at hospital arrival, the detailed time course of resuscitation, survival to hospital discharge, and cerebral performance category at hospital discharge. Initial shockable rhythm was defined as ventricular fibrillation, pulseless ventricular tachycardia, or rhythm for defibrillation in an automated external defibrillator used by emergency medical staff.

### Variables of interest

The variables of interest were body temperature at hospital arrival and low-flow time. Body temperatures were measured at the body surface or core body temperature, which were not recorded in this registry. Following the previous studies on AH [9, 23], we defined AH as body temperatures < 32 °C.

Low-flow time was defined as follows: (i) the duration from cardiac arrest to the establishment of ECPR when the cardiac arrest occurred in the ambulance, (ii) the duration from the call for an ambulance to the establishment of ECPR when the cardiac arrest occurred before ambulance arrival with bystander CPR, or (iii) the duration from the arrival of emergency medical service to the establishment of ECPR when the cardiac arrest occurred before ambulance arrival without bystander CPR.

### Outcomes

The primary outcome was survival at hospital discharge. The secondary outcome was favorable neurological outcome, defined as a cerebral performance category of 1 or 2 at hospital discharge.

### Statistical analysis

Categorical variables were counted and presented as proportions. Continuous variables were expressed as medians and interquartile ranges.

First, we compared the baseline characteristics and outcomes using the Wilcoxon rank-sum test for continuous variables and the Chi-square test for categorical variables.

Next, we examined the non-linear associations between body temperature at hospital arrival and outcomes for all eligible patients using restricted cubic spline analyses [24]. We set four knots in the cubic splines, placed on the fifth, 35th, 65th, and 95th percentile of body temperature [25]. We adjusted for age, sex, the location of cardiac arrest, witnessed cardiac arrest, bystander CPR, the initial cardiac rhythm at the scene and upon hospital arrival and low-flow time. We calculated the adjusted outcomes and their 95% confidence intervals (CIs) for each value of body temperature.

Then, we examined the non-linear associations between low-flow time and outcomes using restricted cubic spline analyses stratified by the presence or absence of AH (hypothermia defined as body temperature below 32 °C). We set four knots in the cubic splines, placed on the fifth, 35th, 65th, and 95th percentiles of low-flow time. We adjusted for age, sex, location of cardiac arrest, witnessed cardiac arrest, bystander CPR, and the initial cardiac rhythm at the scene and upon hospital arrival. We calculated the adjusted outcomes and their 95% CIs for each value of low-flow time.

Finally, we stratified the patients into two groups according to the cut-off value of low-flow time. Since a method has not been universally accepted for determining the cut-off value, we defined a new method in this study to assess the association between low-flow time and outcomes in patients with AH and compared them with those of patients without AH. We defined the cut-off value based on the qualitative assessment of the appearance of the restricted cubic spline curves on the survival discharge rate. Then, we investigated whether the presence of AH had interactions on the association between low-flow time and outcomes by performing multivariable logistic regression analyses for the outcomes with the category of low-flow time (short or long), the presence of AH, their interaction term, and same covariates adjusted in the restricted cubic spline analyses. The odds ratios (ORs) and their 95% CIs on outcomes were calculated for each body temperature group with respect to the reference group with long low-flow time.

As a sensitivity analysis, with the definition of AH as below 28 °C instead of below 32 °C, we examined the non-linear association between low-flow time and outcomes and investigated whether AH had interactions on the association between low-flow time and outcomes in the same way as in the main analysis. AH was defined as a body temperature below 28 °C because the risk of cardiac arrest increases substantially if the core temperature drops below 28 ℃ [1], and severe hypothermia is usually defined as a core temperature below 28 ℃ [26].

Statistical analyses were performed using STATA/BE 17.0 software (StataCorp, College Station, TX, USA) and R 4.3.1 software (R Foundation for Statistical Computing, Vienna, Austria).

## Results

Of the 2,157 adult patients with OHCA who received ECPR, 1,252 were eligible in this analysis. Of these, 105 (8.4%) were in the AH group and 1,147 (91.6%) were in the non-AH group (Additional file 1: Figure S1).

### Characteristics of patients at baseline and outcomes

As to the all eligible patients, the median age was 61 years, and 82.5% were male (Table 1). The median body temperature was 35.1 °C (34.0–35.8 °C), and the median low-flow time was 52 (42–63) min. The overall survival at hospital discharge and favorable neurological outcome were 27.0% and 13.9%, respectively. Patients in the AH group were older, more likely to be female, more likely to be unwitnessed, less likely to receive bystander CPR, and more likely to have initial unshockable rhythm. The median low-flow times, survival at hospital discharge, and favorable neurological outcomes of patients in the AH and non-AH groups were 60 (47–79) and 51 (42–62) min, 44.8% and 25.4%, and 25.7% and 12.8%, respectively (all *p* < 0.001). Only one patient in the AH group had serum potassium levels > 10 mmol/L at hospital arrival (Additional file 2: Table S1).

**Table 1:** Patient characteristics and outcomes of the AH and non-AH groups

Variables	Total	AH	Non-AH	*p* value
	*n* = 1252	*n* = 105	*n* = 1147	
Age, years, median (IQR)	61 (49–69)	66 (56–74)	60 (49–68)	< 0.001
Male, *n* (%)	1033 (82.5%)	76 (72.4%)	957 (83.4%)	0.004
Location of cardiac arrest, *n* (%)				
Home	514 (41.1%)	39 (37.1%)	475 (41.4%)	0.52
Public space	596 (47.6%)	51 (48.6%)	545 (47.5%)	
Ambulance	142 (11.3%)	15 (14.3%)	127 (11.1%)	
Witness, *n* (%)	965 (77.1%)	49 (46.7%)	916 (79.9%)	< 0.001
Bystander CPR, *n* (%)	727 (58.1%)	47 (44.8%)	680 (59.3%)	0.004
Initial cardiac rhythm at the scene, *n* (%)				
Shockable	808 (64.5%)	57 (54.3%)	751 (65.5%)	0.022
Unshockable	444 (35.5%)	48 (45.7%)	396 (34.5%)	
Initial cardiac rhythm at hospital arrival, *n* (%)				
Shockable	598 (47.8%)	53 (50.5%)	545 (47.5%)	0.56
Unshockable	654 (52.2%)	52 (49.5%)	602 (52.5%)	
Body temperature at hospital arrival, °C, median (IQR)	35.1 (34.0–35.8)	26 (22.9–29.8)	35.2 (34.3–35.9)	< 0.001
Low-flow time, minutes, median (IQR)	52 (42–63)	60 (47–79)	51 (42–62)	< 0.001
Outcome, *n* (%)				
Survival at hospital discharge	338 (27.0%)	47 (44.8%)	291 (25.4%)	< 0.001
Favorable neurological outcome	174 (13.9%)	27 (25.7%)	147 (12.8%)	< 0.001

AH: accidental hypothermia; CPR: cardiopulmonary resuscitation; IQR: interquartile range

### Association between body temperature and outcomes

**Fig. 1:**
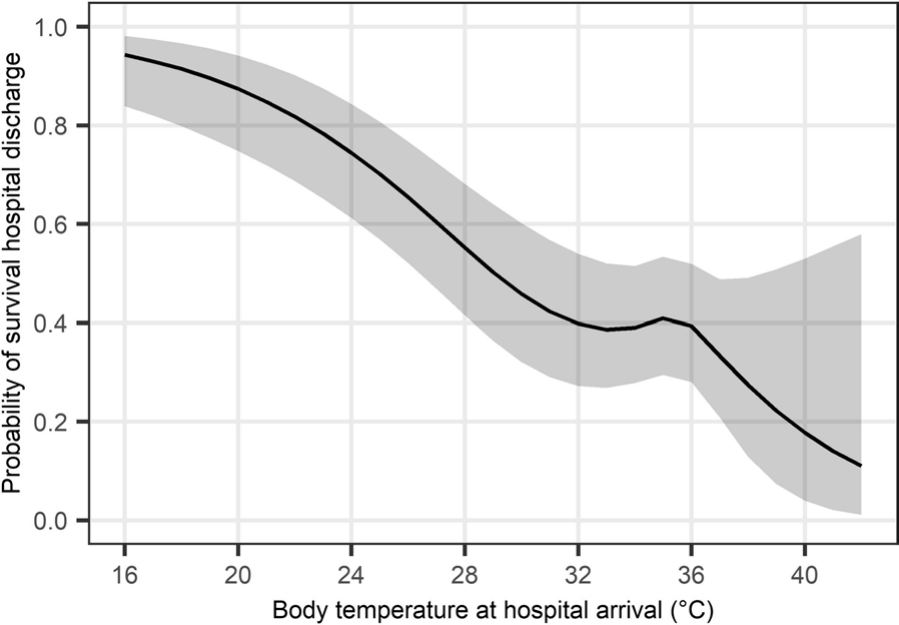
Non-linear associations between arrival body temperature and survival discharge. Four body temperature points (26.7, 34.5, 35.5, and 36.7 °C) were used as the knots in the cubic splines. In the cubic spline analyses, we adjusted for age, sex, location of cardiac arrest, witnessed cardiac arrest, bystander cardiopulmonary resuscitation, the initial cardiac rhythm at the scene and upon hospital arrival, and low-flow time

The cubic spline analysis of survival discharge rate and favorable neurological outcomes of all eligible patients showed increased trend with lower body temperature below 32 °C, with little change between 32 °C and 36 °C (Fig. 1**, **Additional file 3: Figure S2).

### Association between low-flow time and outcomes in the AH and non-AH groups

**Fig. 2:**
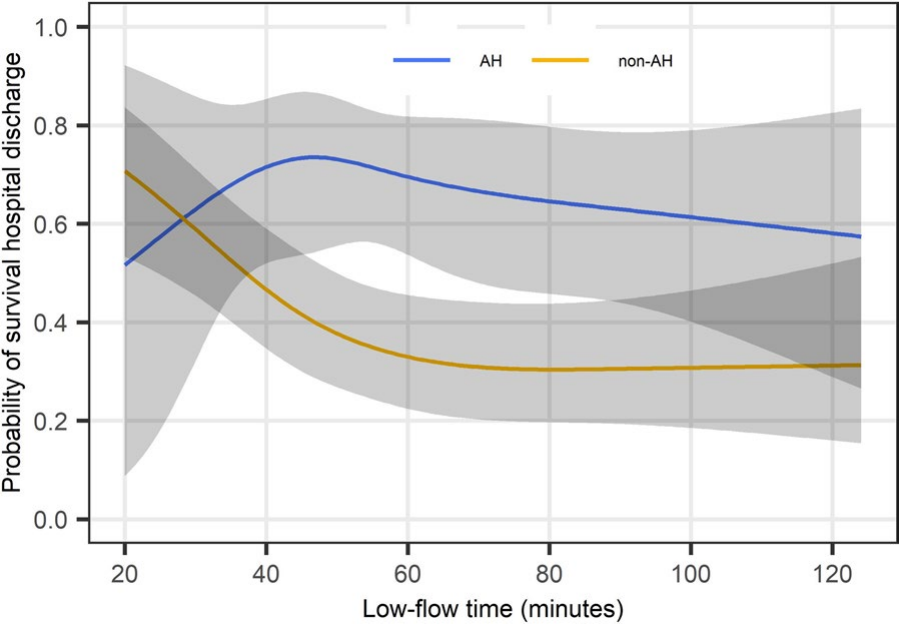
Non-linear associations between low-flow time and survival discharge stratified by the presence of accidental hypothermia. Four low-flow time points (28, 46, 57, and 88 min) were used as the knots in the cubic splines. In the cubic spline analyses, we adjusted for age, sex, location of cardiac arrest, witnessed cardiac arrest, bystander cardiopulmonary resuscitation, and the initial cardiac rhythm at the scene and upon hospital arrival

In the AH group, the cubic spline analysis showed that the survival discharge rate remained relatively unchanged irrespective of the low-flow time **(**Fig. 2). In the non-AH group, the cubic spline analysis showed decreased trend from 20 to 60 min, with little change above 60 min. The survival discharge rate was higher in the AH group between 50 to 90 min of low-flow time compared with that in the non-AH group. The cubic spline analysis on favorable neurological outcomes showed a similar trend (Additional file 4: Figure S3).

Based on the cubic spline curve between the low-flow time and survival outcomes (Fig. 2), patients were stratified into the short (1–50 min) and long (> 50 min) low-flow time groups. In the AH group, the survival discharge rate in the short and long low-flow time groups was 45.5% and 44.4%, respectively, with no statistically significant difference between the groups (OR, 1.05; 95% CI 0.40–2.79). In the non-AH group, the survival discharge rate in the short and long low-flow time groups was 32.1% and 19.2%, respectively, with statistically significant difference between the groups (OR, 0.54; 95% CI 0.40–0.73). The interaction analyses showed a statistically significant interaction between low-flow time and AH group in survival discharge rate (*p* for interaction = 0.048) (Table 2). For the favorable neurological outcome, there was not a significant interaction between low-flow time and the AH group (*p* for interaction = 0.058).

**Table 2:** Outcomes of patients in the AH and non-AH groups with different low-flow times

	Low-flow time		OR (95% CIs)	*p* value	*p* for interaction
	Short (0–50 min)	Long (> 50 min)			
	*n* (%)	*n* (%)			
Survival at hospital discharge					0.048
AH	15/33 (45.5%)	32/72 (44.4%)	1.05 (0.40–2.79)	0.92	
Non-AH	177/552 (32.1%)	114/595 (19.2%)	0.54 (0.40–0.73)	< 0.001	
Favorable neurological outcome					0.058
AH	6/33 (18.2%)	21/72 (29.2%)	1.96 (0.61–6.30)	0.26	
Non-AH	76/552 (13.8%)	71/595 (12.0%)	0.95 (0.65–1.39)	0.79	

AH: accidental hypothermia; CI: confidence interval; OR: odds ratio

### Sensitivity analyses changing threshold of AH to below 28 °C

When patients were stratified according to the body temperature (below and above 28 °C), 72 (5.8%) and 1,180 (94.2%) were included in the AH and non-AH groups, respectively (Additional file 5: Table S2). The cubic spline curve of outcomes and low-flow time was similar to the main results (Additional file 6: Figure S4, Additional file 7: Figure S5). Statistically significant interaction between low-flow time and body temperature was observed for the survival discharge rate (*p* for interaction = 0.027), but not for favorable neurological outcome (*p* for interaction = 0.14) (Additional file 8: Table S3).

## Discussion

This study of adult patients with OHCA who had undergone ECPR revealed an increasing trend in the survival discharge and favorable neurological outcome associated with a lower body temperature at arrival below 32 °C. Moreover, the survival outcome for AH patients with a temperature lower than 32 °C remained constant regardless of low-flow time, and there was a significant interaction of their association with patients without AH.

Previous case reports have shown that even with prolonged low-flow time, hypothermia patients with cardiac arrest who undergo ECPR may survive without neurological impairment [10–12]. However, this is the first observational study to investigate the association between low-flow time and outcomes of cardiac arrest patients with AH who received ECPR. The main cause of death of the patients resuscitated from OHCA is hypoxic–ischemic brain injury [27]. Hypothermia has protective effect toward cerebral cell death caused by circulation insufficiency with decreasing cerebral metabolic activity and oxygen consumption [28–30]. Therefore, it may be pathophysiologically plausible that OHCA patients with AH who received ECPR have good survival outcomes, even with prolonged low-flow time, in contrast to those without AH.

The guidelines from European Resuscitation Council and Extracorporeal Life Support Organization recommend establishing ECPR within 60 min of cardiac arrest without AH [18, 19]. However, this study’s results suggest that different criteria would be applied in the selection of ECPR indications for low-flow time in patients with a body temperature below 32 °C compared to those applicable to patients without hypothermia. In this study, many AH patients had good prognosis when undergoing ECPR, even if the low-flow time to ECPR was as long as 120 min. Therefore, based on our findings, introducing ECPR for AH patients may be reasonable, even if the low-flow time is longer. Further studies are needed to validate our findings.

This study has several limitations. First, although we adjusted the variables as extensively as possible, the large differences in patient characteristics may limit the comparability of the groups because of potential unmeasured confounders, including no-flow time, whether the AH was primary or secondary and the underlying diseases, and whether cardiac arrest preceded the body temperature drop. Concerning the presence of preceding cardiac arrest, serum potassium levels > 10 or 12 mmol/L are considered a marker of existence of hypoxia before body core temperature drop [1]. In this study, only one patient in the AH group had serum potassium levels > 10 mmol/L. Therefore, most AH patients are presumed to have been cooled before cardiac arrest. Second, the methods of temperature measurement were not recorded in this registry; therefore, arrival body temperatures of some patients may be skin temperatures, not core body temperature. In a cold environment, the core body temperature drops slower than skin temperature does [31]. Therefore, we might have misclassified some patients without AH as having AH. However, the survival outcome of non-AH patients was poorer than that of AH patients, and this misclassification caused a bias toward the null hypothesis. Therefore, our results remain robust against this possible bias. Furthermore, an additional questionnaire was administered to examine the details of temperature management in the participating institutions [32]. The questionnaire revealed that > 90% of the institutions monitored the core body temperature. Therefore, the bias due to the absence of a record of the body temperature measurement method in this study was negligible. Third, we approximated low-flow time by calculating the duration from the ambulance call to the initiation of ECPR in cases where cardiac arrest occurred at home or in a public space, and bystander CPR was administered, but this definition did not accurately capture the low-flow time. In cases where telecommunicator CPR was performed, a time lag existed between the emergency call and the initiation of CPR. Therefore, the actual low-flow time might have been longer than the estimated values in these cases. Fourth, the timing of body temperature measurements was not recorded in the registry, and the body temperatures of several patients may have been affected by heat exchangers. However, the measurement of body temperature does not require much time, and the implementation of a heat exchanger is usually performed after admission to the intensive care unit. Therefore, it is reasonable to assume that body temperature was minimally affected by the heat exchanger.

## Conclusions

Adult OHCA patients with arrival body temperature lower than 32 °C who received ECPR had relatively good survival outcome regardless of low-flow time, in contrast to those without AH. In adult OHCA patients with AH, implementing ECPR may be considered even with a prolonged low-flow time.

### Supplementary Information

**Additional file 1: Figure S1.** Patient flowchart. AH, accidental hypothermia; ECMO, extracorporeal membrane oxygenation; ECPR, extracorporeal cardiopulmonary resuscitation; ROSC, return of spontaneous circulation.

**Additional file 2: Table S1.** Serum potassium levels at hospital arrival of patients in the AH group.

**Additional file 3: Figure S2.** Non-linear associations between arrival body temperature and favorable neurological outcome. Four body temperature points (26.7, 34.5, 35.5, and 36.7 °C) were used as the knots in the cubic splines. In the cubic spline analyses, we adjusted for age, sex, location of cardiac arrest, witnessed cardiac arrest, bystander cardiopulmonary resuscitation, the initial cardiac rhythm at the scene and upon hospital arrival, and low-flow time.

**Additional file 4: Figure S3.** Non-linear associations between low-flow time and favorable neurological outcome stratified by the presence of accidental hypothermia. Four low-flow time points (28, 46, 57, and 88 min) were used as the knots in the cubic splines. In the cubic spline analyses, we adjusted for age, sex, location of cardiac arrest, witnessed cardiac arrest, bystander cardiopulmonary resuscitation, and the initial cardiac rhythm at the scene and upon hospital arrival.

**Additional file 5: Table S2.** Patient characteristics and outcomes of the patients with arrival body temperature below and above 28 °C.

**Additional file 6: Figure S4.** Non-linear associations between low-flow time and survival discharge stratified by the body temperature below and above 28 °C. Four low-flow time points (28, 46, 57, and 88 min) were used as the knots in the cubic splines. In the cubic spline analyses, we adjusted for age, sex, location of cardiac arrest, witnessed cardiac arrest, bystander cardiopulmonary resuscitation, and the initial cardiac rhythm at the scene and upon hospital arrival.

**Additional file 7: Figure S5.** Non-linear associations between low-flow time and favorable neurological outcome stratified by the body temperature below and above 28 °C. Four low-flow time points (28, 46, 57, and 88 min) were used as the knots in the cubic splines. In the cubic spline analyses, we adjusted for age, sex, location of cardiac arrest, witnessed cardiac arrest, bystander cardiopulmonary resuscitation, and the initial cardiac rhythm at the scene and upon hospital arrival.

**Additional file 8: Table S3.** Outcomes of patients with arrival body temperature below and above 28 °C with different low-flow times.

## Data Availability

Please contact the author for data and requests.

## Abbreviations

AH: Accidental hypothermia
CI: Confidence intervals
CPR: Cardiopulmonary resuscitation
ECMO: Extracorporeal membrane oxygenation
ECPR: Extracorporeal cardiopulmonary resuscitation
OHCA: Out-of-hospital cardiac arrest
OR: Odds ratio
