# Supplementary figures and images for: Low-flow time and outcomes in hypothermic cardiac arrest patients treated with extracorporeal cardiopulmonary resuscitation: a secondary analysis of a multi-center retrospective cohort study
Source: J Intensive Care. 2024 Jun 11;12:22. doi: 10.1186/s40560-024-00735-1 (PMC11165865; doi:10.1186/s40560-024-00735-1)

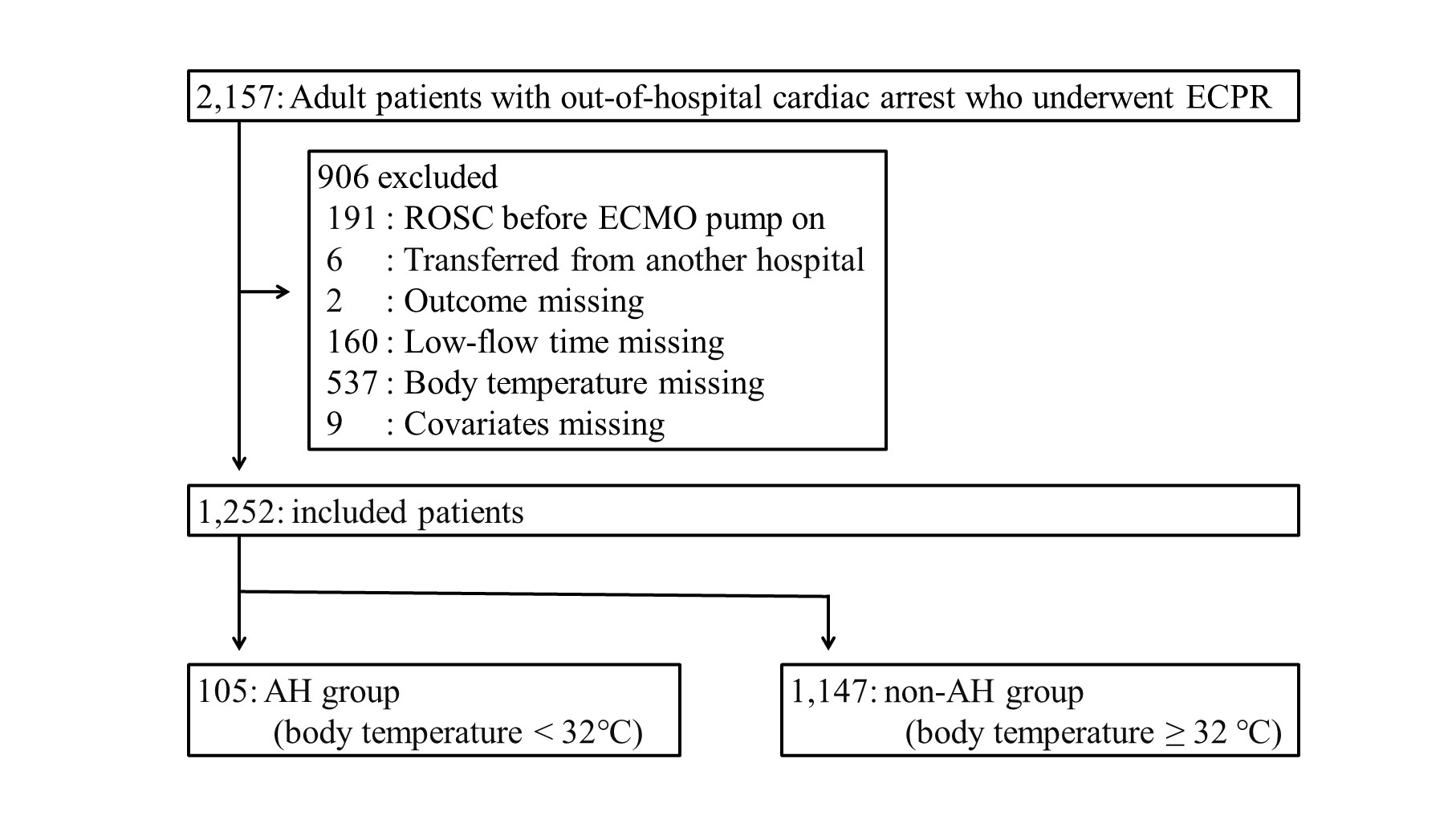

Supplement: Supplementary file 1 — Additional file 1: Figure S1. Patient flowchart. AH, accidental hypothermia; ECMO, extracorporeal membrane oxygenation; ECPR, extracorporeal cardiopulmonary resuscitation; ROSC, return of spontaneous circulation. [file 40560_2024_735_MOESM1_ESM.jpg]

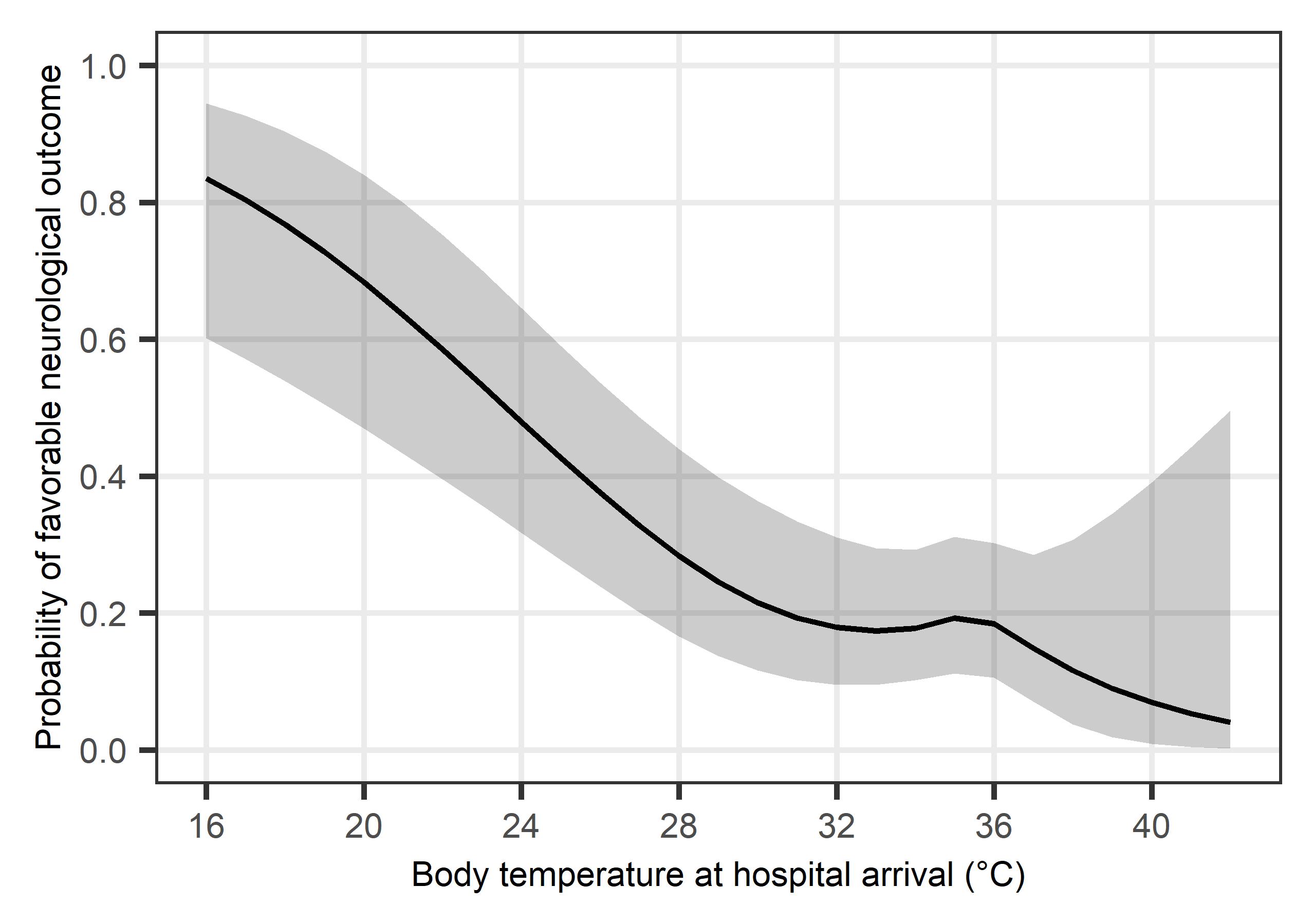

Supplement: Supplementary file 3 — Additional file 3: Figure S2. Non-linear associations between arrival body temperature and favorable neurological outcome. Four body temperature points (26.7, 34.5, 35.5, and 36.7 °C) were used as the knots in the cubic splines. In the cubic spline analyses, we adjusted for age, sex, location of cardiac arrest, witnessed cardiac arrest, bystander cardiopulmonary resuscitation, the initial cardiac rhythm at the scene and upon hospital arrival, and low-flow time. [file 40560_2024_735_MOESM3_ESM.jpeg]

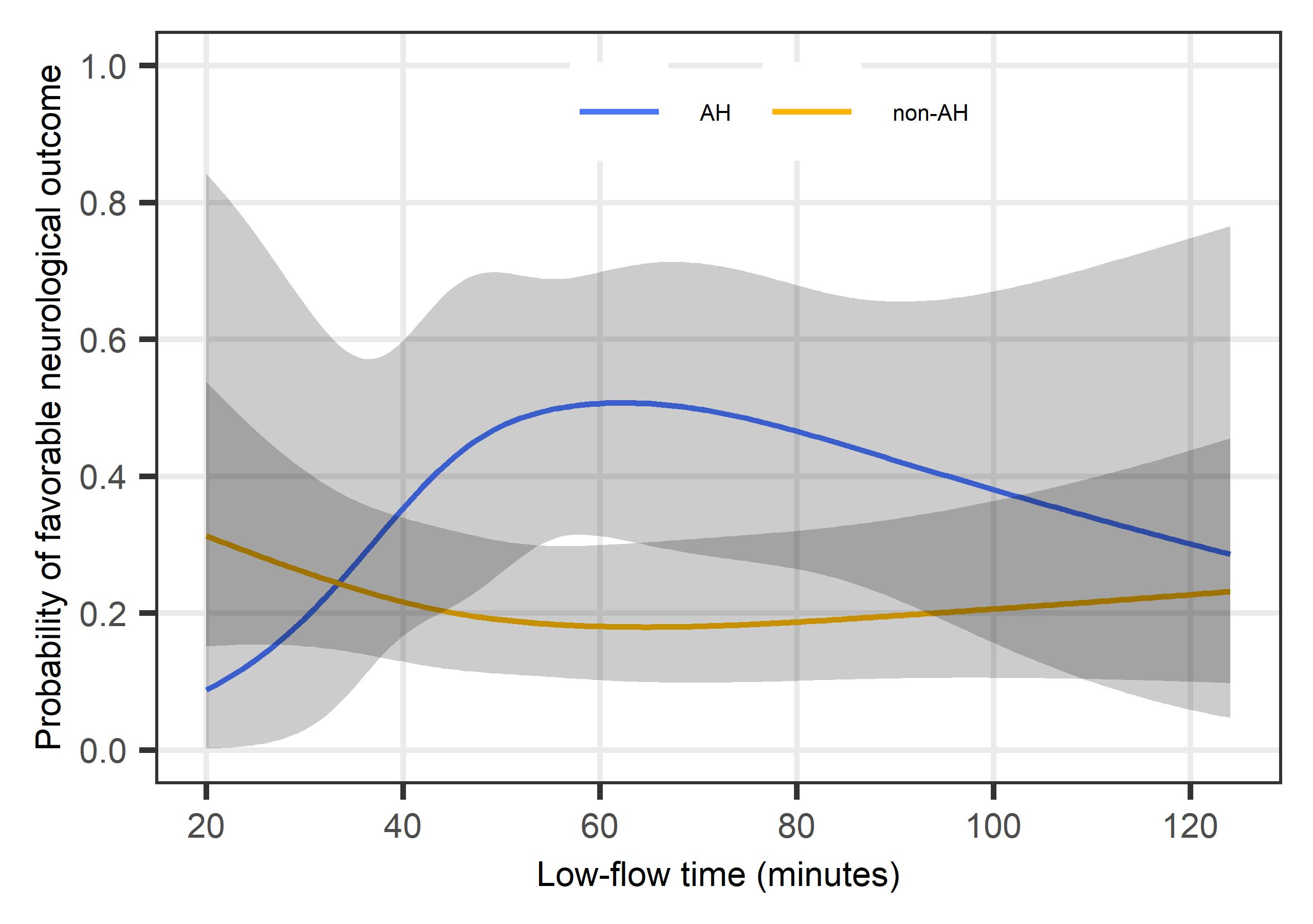

Supplement: Supplementary file 4 — Additional file 4: Figure S3. Non-linear associations between low-flow time and favorable neurological outcome stratified by the presence of accidental hypothermia. Four low-flow time points (28, 46, 57, and 88 min) were used as the knots in the cubic splines. In the cubic spline analyses, we adjusted for age, sex, location of cardiac arrest, witnessed cardiac arrest, bystander cardiopulmonary resuscitation, and the initial cardiac rhythm at the scene and upon hospital arrival. [file 40560_2024_735_MOESM4_ESM.jpeg]

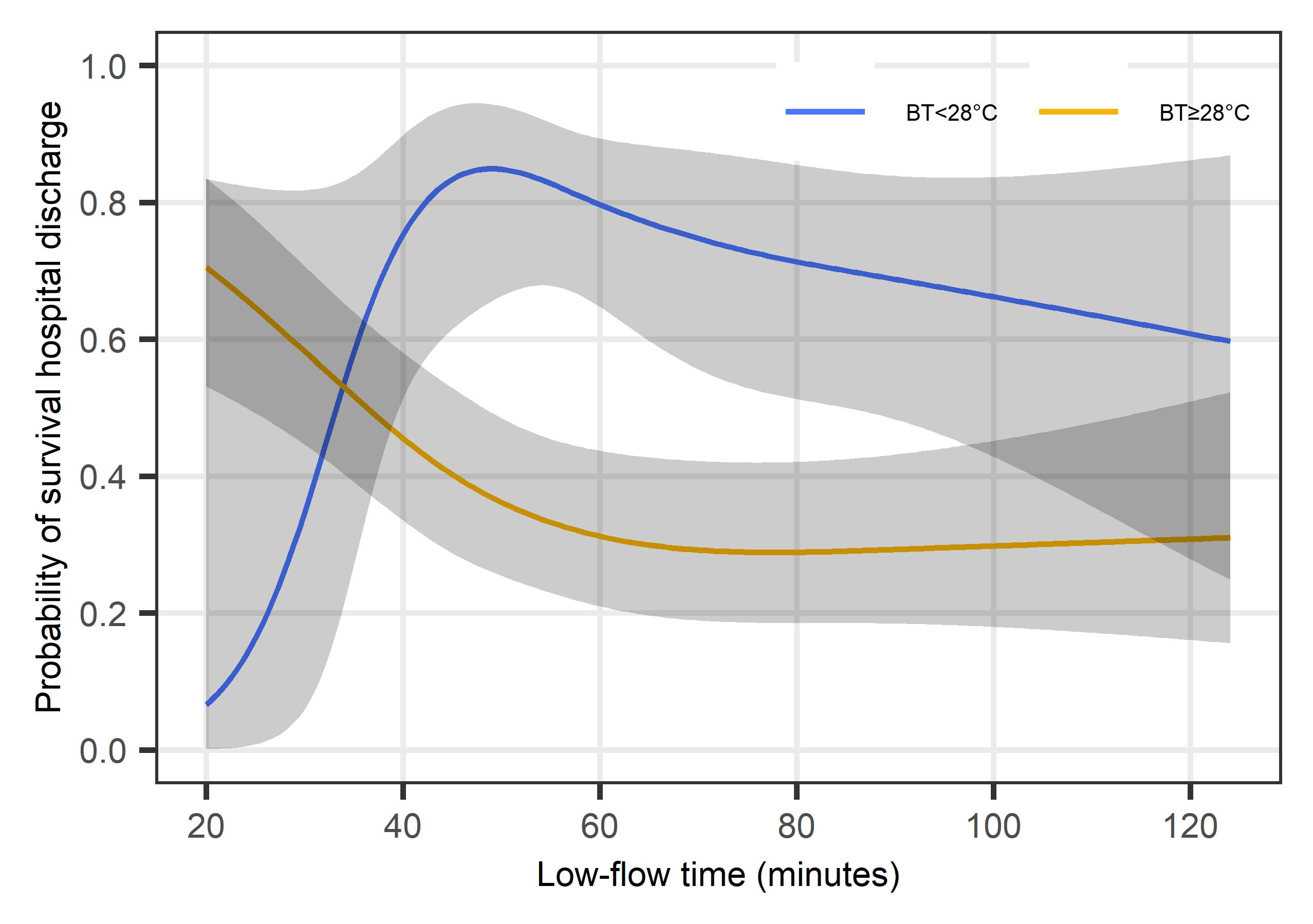

Supplement: Supplementary file 6 — Additional file 6: Figure S4. Non-linear associations between low-flow time and survival discharge stratified by the body temperature below and above 28 °C. Four low-flow time points (28, 46, 57, and 88 min) were used as the knots in the cubic splines. In the cubic spline analyses, we adjusted for age, sex, location of cardiac arrest, witnessed cardiac arrest, bystander cardiopulmonary resuscitation, and the initial cardiac rhythm at the scene and upon hospital arrival. [file 40560_2024_735_MOESM6_ESM.jpeg]

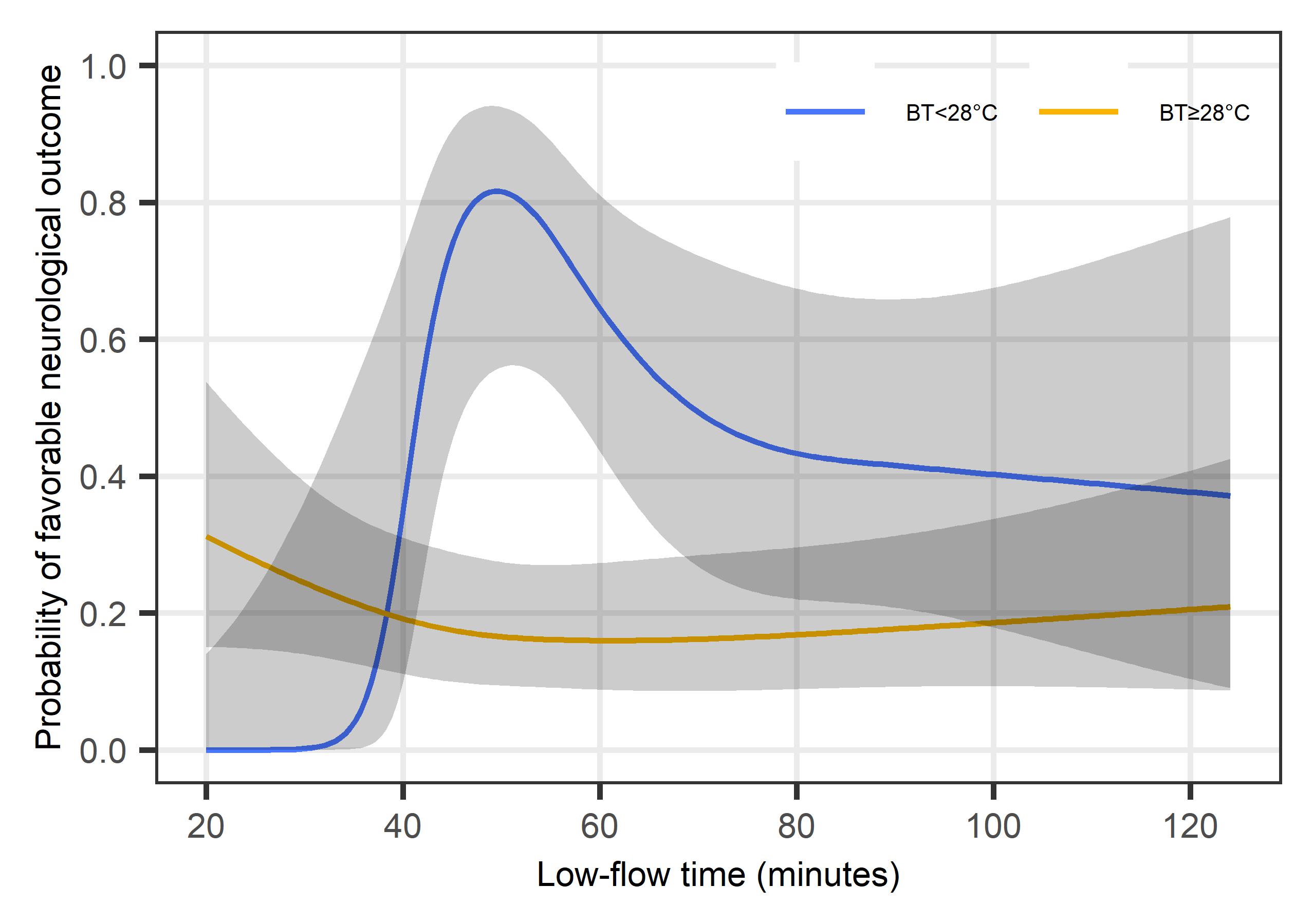

Supplement: Supplementary file 7 — Additional file 7: Figure S5. Non-linear associations between low-flow time and favorable neurological outcome stratified by the body temperature below and above 28 °C. Four low-flow time points (28, 46, 57, and 88 min) were used as the knots in the cubic splines. In the cubic spline analyses, we adjusted for age, sex, location of cardiac arrest, witnessed cardiac arrest, bystander cardiopulmonary resuscitation, and the initial cardiac rhythm at the scene and upon hospital arrival. [file 40560_2024_735_MOESM7_ESM.jpeg]
