# Supplementary material for: Low-flow time and outcomes in hypothermic cardiac arrest patients treated with extracorporeal cardiopulmonary resuscitation: a secondary analysis of a multi-center retrospective cohort study
Source: J Intensive Care. 2024 Jun 11;12:22. doi: 10.1186/s40560-024-00735-1 (PMC11165865; doi:10.1186/s40560-024-00735-1)
Supplement: Supplementary file 2 — Additional file 2: Table S1. Serum potassium levels at hospital arrival of patients in the AH group. [file 40560_2024_735_MOESM2_ESM.docx]

**Table S1. Serum potassium levels at hospital arrival of patients in the AH group**

| Serum potassium level (mmol/L) | Number of patients | Cumulative proportion (%) |
| --- | --- | --- |
| 2.08 | 1 | 0.95 |
| 2.42 | 1 | 1.9 |
| 2.77 | 1 | 2.86 |
| 2.80 | 1 | 3.81 |
| 2.82 | 1 | 4.76 |
| 2.86 | 1 | 5.71 |
| 2.90 | 2 | 7.62 |
| 2.99 | 1 | 8.57 |
| 3.00 | 1 | 9.52 |
| 3.07 | 1 | 10.48 |
| 3.1 | 1 | 11.43 |
| 3.11 | 1 | 12.38 |
| 3.13 | 1 | 13.33 |
| 3.30 | 3 | 16.19 |
| 3.34 | 1 | 17.14 |
| 3.37 | 1 | 18.1 |
| 3.46 | 1 | 19.05 |
| 3.5 | 1 | 20 |
| 3.53 | 1 | 20.95 |
| 3.58 | 1 | 21.9 |
| 3.60 | 5 | 26.67 |
| 3.67 | 1 | 27.62 |
| 3.70 | 2 | 29.52 |
| 3.73 | 1 | 30.48 |
| 3.80 | 1 | 31.43 |
| 3.83 | 1 | 32.38 |
| 3.87 | 1 | 33.33 |
| 3.90 | 2 | 35.24 |
| 3.96 | 1 | 36.19 |
| 4.00 | 2 | 38.1 |
| 4.01 | 1 | 39.05 |
| 4.10 | 3 | 41.9 |
| 4.16 | 1 | 42.86 |
| 4.20 | 2 | 44.76 |
| 4.25 | 1 | 45.71 |
| 4.30 | 5 | 50.48 |
| 4.40 | 1 | 51.43 |
| 4.45 | 2 | 53.33 |
| 4.47 | 1 | 54.29 |
| 4.60 | 1 | 55.24 |
| 4.61 | 1 | 56.19 |
| 4.67 | 1 | 57.14 |
| 4.69 | 1 | 58.1 |
| 4.70 | 1 | 59.05 |
| 4.80 | 2 | 60.95 |
| 4.85 | 1 | 61.9 |
| 4.9 | 1 | 62.86 |
| 4.93 | 1 | 63.81 |
| 5.00 | 2 | 65.71 |
| 5.05 | 1 | 66.67 |
| 5.10 | 2 | 68.57 |
| 5.21 | 1 | 69.52 |
| 5.30 | 1 | 70.48 |
| 5.34 | 1 | 71.43 |
| 5.40 | 2 | 73.33 |
| 5.45 | 1 | 74.29 |
| 5.53 | 1 | 75.24 |
| 5.60 | 1 | 76.19 |
| 5.72 | 1 | 77.14 |
| 5.87 | 1 | 78.1 |
| 5.90 | 1 | 79.05 |
| 6.00 | 1 | 80 |
| 6.07 | 2 | 81.9 |
| 6.10 | 1 | 82.86 |
| 6.20 | 1 | 83.81 |
| 6.43 | 1 | 84.76 |
| 6.60 | 2 | 86.67 |
| 6.80 | 1 | 87.62 |
| 6.90 | 1 | 88.57 |
| 7.55 | 1 | 89.52 |
| 7.73 | 1 | 90.48 |
| 7.80 | 2 | 92.38 |
| 8.40 | 1 | 93.33 |
| 8.70 | 1 | 94.29 |
| 10.5 | 1 | 95.24 |
| missing | 5 |  |
