# Supplementary material for: Low-flow time and outcomes in hypothermic cardiac arrest patients treated with extracorporeal cardiopulmonary resuscitation: a secondary analysis of a multi-center retrospective cohort study
Source: J Intensive Care. 2024 Jun 11;12:22. doi: 10.1186/s40560-024-00735-1 (PMC11165865; doi:10.1186/s40560-024-00735-1)
Supplement: Supplementary file 5 — Additional file 5: Table S2. Patient characteristics and outcomes of the patients with arrival body temperature below and above 28 °C. [file 40560_2024_735_MOESM5_ESM.docx]

**Table S2. Patient characteristics and outcomes of the patients with arrival body temperature below and above 28°C**

|  | Total | Arrival body temperature <28°C | Arrival body temperature >=28°C | p-value |
| --- | --- | --- | --- | --- |
| Variables | n=1,252 | n=72 | n=1,180 |  |
| Age, years, median (IQR) | 61 (49-69) | 66 (54.5-78) | 60 (49-68) | <0.001 |
| Male, n (%) | 1,033 (82.5%) | 47 (65.3%) | 986 (83.6%) | <0.001 |
| Location of cardiac arrest, n (%) |  |  |  |  |
| Home | 514 (41.1%) | 26 (36.1%) | 488 (41.4%) | 0.081 |
| Public space | 596 (47.6%) | 32 (44.4%) | 564 (47.8%) |  |
| Ambulance | 142 (11.3%) | 14 (19.4%) | 128 (10.8%) |  |
| Witness, n (%) | 965 (77.1%) | 28 (38.9%) | 937 (79.4%) | <0.001 |
| Bystander CPR, n (%) | 727 (58.1%) | 32 (44.4%) | 695 (58.9%) | 0.016 |
| Initial cardiac rhythm at the scene, n (%) |  |  |  |  |
| Shockable | 808 (64.5%) | 36 (50.0%) | 772 (65.4%) | 0.008 |
| Unshockable | 444 (35.5%) | 36 (50.0%) | 408 (34.6%) |  |
| Initial cardiac rhythm at hospital arrival, n (%) |  |  |  |  |
| Shockable | 598 (47.8%) | 41 (56.9%) | 557 (47.2%) | 0.11 |
| Unshockable | 654 (52.2%) | 31 (43.1%) | 623 (52.8%) |  |
| Body temperature at hospital arrival, °C, median (IQR) | 35.1 (34.0-35.8) | 24 (22-26.1) | 35.2 (34.2-35.9) | <0.001 |
| Low-flow time, minutes, median (IQR) | 52 (42-63) | 61 (48.5-80.5) | 51 (42-63) | <0.001 |
| Outcome, n (%) |  |  |  |  |
| Survival at hospital discharge | 338 (27.0%) | 38 (52.8%) | 300 (25.4%) | <0.001 |
| Favorable neurological outcome | 174 (13.9%) | 25 (35.2%) | 149 (12.6%) | <0.001 |

IQR, interquartile range; CPR cardiopulmonary resuscitation
