# Supplementary material for: Low-flow time and outcomes in hypothermic cardiac arrest patients treated with extracorporeal cardiopulmonary resuscitation: a secondary analysis of a multi-center retrospective cohort study
Source: J Intensive Care. 2024 Jun 11;12:22. doi: 10.1186/s40560-024-00735-1 (PMC11165865; doi:10.1186/s40560-024-00735-1)
Supplement: Supplementary file 8 — Additional file 8: Table S3. Outcomes of patients with arrival body temperature below and above 28 °C with different low-flow times. [file 40560_2024_735_MOESM8_ESM.docx]

**Table S3. Outcomes of patients with arrival body temperature below and above 28°C with different low-flow time**

|  | low-flow time | |  |  |  |
| --- | --- | --- | --- | --- | --- |
|  | short (0-50 minutes) | long (>50 minutes) |  |  |  |
|  | n (%) | n(%) | OR (95% CIs) | p-value | p for interaction |
| Survival at hospital discharge |  |  |  |  | 0.027 |
| Arrival body temperature <28°C | 9/19 (47.3 %) | 29/53 (54.7 %) | 1.39 (0.37 to 5.25) | 0.63 |  |
| Arrival body temperature >=28°C | 183/566 (32.3 %) | 117/614 (19.0 %) | 0.54 (0.41 to 0.72) | <0.001 |  |
| Favorable neurological outcome |  |  |  |  | 0.14 |
| Arrival body temperature <28°C | 5/19 (26.3 %) | 20/53 (37.7 %) | 1.02 (0.25 to 4.19) | 0.98 |  |
| Arrival body temperature >=28°C | 77/566 (13.6 %) | 72/614 (11.7 %) | 0.96 (0.66 to 1.40) | 0.83 |  |

OR, odds ratio; CI, confidence interval
